# In Vivo Evaluation of ^68^Ga-Labeled NOTA-EGFRvIII Aptamer in EGFRvIII-Positive Glioblastoma Xenografted Model

**DOI:** 10.3390/pharmaceutics16060814

**Published:** 2024-06-16

**Authors:** Jun Young Park, Ye Lim Cho, Tae Sup Lee, Daekyun Lee, Ju-Hyung Kang, Soryong Lim, Yujin Lee, Jae Hyun Lim, Won Jun Kang

**Affiliations:** 1 Department of Nuclear Medicine, Severance Hospital, Yonsei University College of Medicine, 50-1 Yonsei-ro, Seodaemun-gu, Seoul 03722, Republic of Korea; abies60@naver.com (J.Y.P.); etommi@yuhs.ac (Y.L.C.); 2 Division of RI Application, Korea Institute of Radiological and Medical Science (KIRAMS), Seoul 01812, Republic of Korea; nobelcow@kirams.re.kr (T.S.L.); longinolim@nate.com (J.H.L.); 3 Aptamer Sciences Inc., Pangyo Seven Venture Valley 1 (3-dong), 15, Pangyo-ro 228 beon-gil, Bundang-gu, Seongnam-si 13487, Republic of Korea; d.lee@aptsci.com (D.L.); jhkang86@aptsci.com (J.-H.K.); srl94@aptsci.com (S.L.); leeyj@aptsci.com (Y.L.)

**Keywords:** aptamer, EGFRvIII, Ga-68, PET imaging, glioblastoma

## Abstract

EGFRvIII is expressed only in tumor cells and strongly in glioblastoma and is considered a promising target in cancer diagnosis and therapy. Aptamers are synthetic single-stranded oligonucleotides that bind to biochemical target molecules with high binding affinity and specificity. This study examined the potential of the ^68^Ga-NOTA-EGFRvIII aptamer as a nuclear imaging probe for visualizing EGFRvIII-expressing glioblastoma by positron emission tomography (PET). EGFRvIII aptamer was selected using the SELEX technology, and flow cytometry and fluorescence microscopy verified the high binding affinity to EGFRvIII positive U87MG vIII 4.12 glioma cells but not to EGFRvIII negative U87MG cells. The EGFRvIII aptamer was conjugated with a chelator (1,4,7-triazanonane-1,4,7-triyl)triacetic acid (NOTA) for ^68^Ga-labeling. The ^68^Ga-NOTA-EGFRvIII aptamer was prepared using the preconcentration-based labeling method with a high radiolabeling yield at room temperature. Ex vivo biodistribution analyses confirmed the significantly higher tumor uptake of the ^68^Ga-NOTA-EGFRvIII aptamer in EGFRvIII-expressing xenograft tumors than that in EGFRvIII negative tumors, confirming the specific tumor uptake of the ^68^Ga-NOTA-EGFRvIII aptamer in vivo. PET imaging studies revealed a high retention rate of the ^68^Ga-NOTA-EGFRvIII aptamer in U87MG vIII 4.12 tumors but only low uptake levels in U87-MG tumors, suggesting that the ^68^Ga-NOTA-EGFRvIII aptamer may be used as a PET imaging agent for EGFRvIII-expressing glioblastoma.

## 1. Introduction

Glioblastoma is the most common and highly aggressive type of primary brain tumor in adults [1]. Unfortunately, glioblastoma is still considered an incurable brain cancer with a median overall survival of 12.1 months and a five-year survival rate of 6.8% owing to the frequent recurrence and tumor progression [2,3]. Glioblastoma is presumed to arise from neural stem cells in the subventricular zone, resulting in extensive cellular heterogeneity and complexity [4]. Neural stem cells can self-renew and differentiate into different lineages, including neurons, astrocytes, and oligodendrocytes, contributing to the preferential resistance to chemotherapy and radiotherapy [5,6].

The epidermal growth factor receptor (EGFR), a member of the ErbB family of tyrosine kinase receptors, plays an essential role in the receptor-mediated signal transduction involved in regulating cell growth, proliferation, differentiation, migration, and the inhibition of apoptosis [7]. The *EGFR* gene is frequently amplified and mutated in primary glioblastomas, leading to the strong expression of the EGFR protein on the cellular plasma membrane. The most common form of EGFR mutation in glioblastomas is variant III (EGFRvIII), which is characterized by the intragenic deletion of exons 2–7, resulting in the loss of 267 amino acids in the extracellular domain of the receptor [8]. Compared to the wild-type EGFR, EGFRvIII lacks ligand binding activity but is constitutively active without EGF binding owing to mutation-induced conformational changes in the receptor [9]. EGFRvIII has also been detected in non-small-cell lung and breast cancers but not in the corresponding normal tissues. Therefore, EGFRvIII is a promising target for cancer diagnosis and therapy [10].

Aptamers are chemically synthetic single-stranded oligonucleotides that fold up into unique three-dimensional structures with high binding affinity and specificity to various target molecules, including ions, peptides, proteins, viruses, bacteria, small molecules, and whole living cells [11,12]. Aptamers are used extensively in many fields owing to their advantages, including no batch-to-batch variation, the ease of synthesis, minor immunogenicity, versatile chemical modification, and high physical stability [13]. The first aptamers selected against the EGFR family was A30. The RNA aptamer A30 binds to the extracellular domain of EGFR3 and blocks the interactions of EGFR3 with its ligand heregulin, leading to the growth inhibition of MCF7 cells [14]. Anti-EGFR aptamer, CL4, exhibits high binding affinity to the extracellular domain of human EGFR and binds specifically and internalizes to the EGFRvIII-expressing glioma cells [15,16]. The DNA aptamer U2 selected against U87MG-EGFRvIII cells binds specifically to the EGFRvIII protein expressed on the cell surface with high affinity. The rhenium-188-labeled U2 aptamer exhibits specific tumor uptake in U87MG-EGFRvIII xenograft tumors in vivo [17]. Thus far, several aptamers against EGFR have been developed and explored for molecular imaging and targeted drug delivery system, but few studies have reported the in vivo imaging and biodistribution of aptamers against EGFRvIII.

In this current study, a nuclease-resistant idT-containing DNA aptamer against human EGFRvIII protein was generated, and aptamer-based nuclear imaging probes were developed to evaluate EGFRvIII expression in cancer. The bifunctional chelator (1,4,7-triazanonane-1,4,7-triyl)triacetic acid (NOTA) was selected as a chelator and NOTA-conjugated EGFRvIII (NOTA-EGFRvIII) aptamer was synthesized and labeled with the positron-emitting radionuclide gallium-68 (^68^Ga). The targeting properties of ^68^Ga-NOTA-EGFRvIII aptamer were evaluated in vitro and in vivo in mice bearing EGFRvIII-expressing U87MG glioblastoma xenografts.

## 2. Materials and Methods

### 2.1. Materials

All the chemicals and solvents were purchased from Sigma-Aldrich (St. Louis, MI, USA) or Merck (Darmstadt, Germany) and used without further purification. Isothiocyanate benzyl-NOTA *(p*-SCN-Bn-NOTA) was obtained from Macrocyclics, Inc. (Dallas, TX, USA). The IGG-100 germanium-68/gallium-68 (^68^Ge/^68^Ga) generator was acquired from Eckert & Ziegler Radiopharma GmbH (1.85 GBq, Berlin, Germany). The Chromafix^®^ 30-PS-HCO_3_ anion-exchange cartridge was purchased from Macherey-Nagel (Duren, Germany). Radio thin-layer chromatography (TLC) was performed using glass microfiber chromatography paper impregnated with silica gel (iTLC-SG, Agilent Technologies, Santa Clara, CA, USA). The ^68^Ga activities were measured in a CRC^®^-25 PET dose calibrator (Capintec, Florham Park, NJ, USA).

### 2.2. Cell Culture

The human glioblastoma cell line U87MG (Cat# HTB-14™) was obtained from the American Type Culture Collection (Manassas, VA, USA). The DKMG/EGFRvIII cell line (Cat# CL 01008-CLTH) and U87MG vIII 4.12 clone cell line (Cat# CL 01004-CLTH) were purchased from Celther Polska (Lodz, Poland). The U87MG cell line was cultured in Dulbecco’s Modified Eagle’s Medium (DMEM, Gibco, Thermo Fisher Scientific, Waltham, MA, USA) supplemented with 10% fetal bovine serum (FBS, Gibco, Thermo Fisher Scientific), 100 U/mL penicillin, and 100 mg/mL streptomycin (Gibco, Thermo Fisher Scientific). The DKMG/EGFRvIII cell line was cultured in Roswell Park Memorial Institute (RPMI)-1640 (Gibco, Thermo Fisher Scientific) supplemented with 2 mM L-glutamine, 1 mM sodium pyruvate, and 10% FBS. The U87MG vIII 4.12 cell line was cultured in DMEM GlutaMAX streptomycin (Gibco, Thermo Fisher Scientific), supplemented with 10% FBS, 100 U/mL penicillin, 100 mg/mL streptomycin, and 100 μg/mL of G418 disulfate salt (Cat# A1720; Sigma-Aldrich). All the cell lines were maintained at 37 °C in a humidified atmosphere containing 5% CO_2_.

### 2.3. Preparation of EGFRvIII Aptamer

The EGFRvIII aptamers were selected using the Systematic Evolution of Ligands by Exponential enrichment (SELEX) technology from Aptamer Sciences Inc. (Seongnam-si, Republic of Korea). Briefly, the modified single-strand DNA library with a 40-nucleotide random sequence (N40) containing 5-[N-(2-naphthylmethyl)carboxamide]-2′-deoxyuridine (2Nap-dU) or 5-(N-benzylcarboxamide)-2′-deoxyuridine (Bn-dU) instead of the deoxythymidine was prepared as template sequence. The target EGFRvIII protein was immobilized to the magnetic beads and a polymerase chain reaction was performed with the following primer sequence: (forward) 5′-CGA GCG TCC TGC CTT TG-3′, (reverse) 5′-CT GGG TGG CTG TCG GTG-3′. After each round of SELEX, binding assays were performed to measure the equilibrium dissociation constant (K_d_) of each candidate aptamer. The EGFRvIII aptamers were selected from the 2Nap-dU-contained ssDNA library. The full-length EGFRvIII aptamers (74 nucleotides) were truncated into 44 nucleotides based on the secondary structures predicted by the RNAstructure web server to minimize and optimize the aptamer except for the binding domain [18]. The sequence of the EGFRvIII aptamer was 5′-TGA GPA AGP GAG PAC CGP PPG CGA PPP GGA GAA CPA CGC AAP CA-3′ (P represents 2Nap-dU), and inverted deoxythymidine (idT) was incorporated at the end of the EGFRvIII aptamer. The fully optimized EGFRvIII aptamer was conjugated with various materials, such as biotin and NOTA, depending on the needs of the experimental methods.

### 2.4. Western Blot Analysis

The glioblastoma cell lines were harvested and lysed with RIPA Lysis and Extraction Buffer (Thermo Fisher Scientific, Waltham, MA, USA) containing Halt™ Protease Inhibitor Cocktail (Thermo Fisher Scientific) on ice and centrifuged at 14,000× *g* for 20 min at 4 °C. The protein samples were quantified using the Pierce™ BCA Protein Assay Kit (Cat# 23225; Thermo Fisher Scientific). Equal amounts of protein (20 µg) were separated on 10% sodium dodecyl sulfate polyacrylamide gel electrophoresis and transferred onto polyvinylidene fluoride (PVDF) membranes (Bio-Rad Laboratories, Hercules, CA, USA). The membranes were blocked with 5% skim milk in Tris-buffered saline containing 0.1% Tween-20 (TBST) for 1 h at room temperature and then incubated with the primary antibodies against EGFRvIII (Cat# Ab00184-1.4; Absolute Antibody, San Diego, CA, USA) overnight at 4 °C at a 1:1000 dilution. The membranes were washed three times with TBST and incubated with horseradish peroxidase-conjugated secondary antibodies (m-IgGκ BP-HRP, Cat# SC-516102; Santa Cruz Biotechnology, Santa Cruz, CA, USA). The protein bands were detected using the Pierce™ ECL Plus Western Blotting Substrate (Thermo Scientific). The integrated optical density for the protein band was captured and quantified using ChemiDoc XRS+ imaging systems (Bio-Rad Laboratories).

### 2.5. Flow Cytometry

The expression of the EGFRvIII protein on the surface of cells was verified by flow cytometry on the U87MG, DKMG/EGFRvIII, and U87MG vIII 4.12 cells. In total, 5 × 10^5^ cells were incubated with 2 µg of FITC-labeled anti-EGFRvIII antibody (Cat# NBP2-50599F; Novus Biologicals, Centennial, CO, USA) or the isotype control antibody (Cat# ab106163; Abcam, Cambridge, MA, USA) for 60 min at 4 °C on a Thermomixer (Eppendorf AG, Hamburg, Germany) in FACS buffer (PBS containing 1% BSA). After washing with FACS buffer, flow cytometric analyses were performed using a FACSCalibur™ (Becton Dickinson, Franklin Lakes, NJ, USA).

The binding affinity of the selected EGFRvIII aptamer with the target cells was also performed using flow cytometry. The U87MG, DKMG/EGFRvIII, and U87MG vIII 4.12 cells were incubated with 100 pmol of Cy5-EGFRvIII aptamer at 4 °C for 30 min in a binding buffer. After washing with an ice-cold binding buffer, flow cytometric analyses were performed using an LSR II flow cytometer (Becton Dickinson).

### 2.6. Confocal Fluorescence Microscopy

The U87MG, DKMG/EGFRvIII, and U87MG vIII 4.12 cells were grown on glass coverslips at a 5 × 10^5^ density and incubated with 100 pmol of Cy5-EGFRvIII or Cy5-scrambled EGFRvIII (ScrEGFRvIII) aptamers in binding buffer (Dulbecco’s phosphate-buffered saline supplemented with 4.5 g/L glucose, 5 mM MgCl_2_, 0.1 mg/mL yeast tRNA, and 1 mg/mL bovine serum albumin) at 4 °C for 30 min. The cells were washed with an ice-cold binding buffer and fixed with 4% paraformaldehyde. The cells were stained with 4′,6-diamidino-2-phenylindole (DAPI, Vector Laboratories, Burlingame, CA, USA) and visualized using a Zeiss LSM-700 confocal microscope (Carl Zeiss, Oberkochen, Germany). Imaging analysis was performed using the ZEN 2010 image software (version 3.3, Zeiss).

### 2.7. Preparation of NOTA Conjugated EGFRvIII Aptamer

The NOTA-EGFRvIII aptamer was synthesized using the *p*-SCN-Bn-NOTA and amine-functionalized EGFRvIII aptamer according to the method described previously [19]. Briefly, 20 nmol of 5′-amine-modified EGFRvIII aptamer was dissolved in sodium tetraborate (pH 9.3). Subsequently, 100 equivalents of *p*-SCN-Bn-NOTA in dimethylformamide were added to the aptamer solution and stirred overnight at room temperature. The reaction mixture was purified using a 3 kDa Amicon spin column (Merck Millipore, Burlington, MA, USA) and concentrated under vacuum to obtain the NOTA-EGFRvIII aptamer (yield; 75%). The conjugation was monitored by urea polyacrylamide gel electrophoresis (urea-PAGE) using 15% polyacrylamide gels containing 8 M urea. The 20/100 DNA Ladder (Integrated DNA Technologies, Coralville, IA, USA) was used as an oligonucleotide length standard. Gels were stained with Gel Star (Lonza, Basel, Switzerland) and imaged with a gel imaging system.

### 2.8. Biolayer Interferometry

The binding affinity of the EGFRvIII aptamers was determined by biolayer interferometry (BLI) using a GatorPrime (Gator Bio, Palo Alto, CA, USA) with a streptavidin (SA) probe (Gator Bio). The biotinylated EGFRvIII aptamers were immobilized on the SA probe. After washing, the SA probe combined with the biotinylated EGFRvIII aptamer was associated with 25–100 nM of the EGFRvIII protein (ACRO Biosystems, Cambridge, MA, USA) for 200 s, followed by a dissociation step of up to 400 s. The association and dissociation curves were graphed and calculated using the GatorOne software (version 2.13.5.0830, Gator Bio) to yield the K_d_ values. The binding affinity of NOTA-EGFRvIII was determined by reacting the nickel-chelated nitrilotriacetate (Ni-NTA) probe (Gator Bio) with the EGFRvIII protein for 10 min at room temperature. The protein-combined Ni-NTA probe was reacted with various concentrations (125, 250, 500 nM) of the NOTA-EGFRvIII aptamer for 200 s, and the Ni-NTA probe-bound aptamers were then dissociated for 400 s in PBS containing 0.05% Tween-20. The K_d_ values were calculated using the GatorOne software (Gator Bio).

### 2.9. Preparation of ^68^Ga-NOTA-EGFRvIII Aptamer

The ^68^GaCl_3_ was concentrated on a Chromafix 30-PS-HCO_3_ anion-exchange cartridge [20]. A 30-PS-HCO_3_ cartridge was pre-activated with 1 mL of a 30% hydrochloric acid solution (HCl, Suprapur^®^ for trace analysis, Merck), followed by washing with 10 mL of deionized/distilled water (ddH_2_O) prior to use. The ^68^GaCl_3_ was eluted from the ^68^Ge/^68^Ga generator with 5 mL of 0.1 N HCl and mixed with 4 mL of 30% HCl. The ^68^Ga solution was loaded on the 30-PS-HCO_3_ cartridge and washed with 1 mL of 5 M HCl. The cartridge was then purged with air to remove any traces of HCl, followed by the slow elution of concentrated ^68^Ga with 0.3 mL of ddH_2_O. The preconcentrated ^68^Ga^3+^ eluate was buffered with 0.25 M sodium acetate and mixed with the NOTA-EGFRvIII aptamer. The final pH of the mixture was adjusted to 4.0–4.2. The reaction mixture was incubated for 10 min at room temperature with gentle shaking. The labeling yields were determined by iTLC-SG using a 1 M ammonium acetate in water/methanol (50/50 *v*/*v*) as the mobile phase. The radioactivity distribution on the iTLC-SG plates was analyzed using an AR2000 radio-TLC imaging scanner (Eckert & Ziegler Radiopharma GmbH). The free ^68^Ga remained at the origin, and the ^68^Ga-NOTA-EGFRvIII aptamer migrated with the solvent front.

### 2.10. Animal Model

Athymic nude mice (seven weeks old) were purchased from Orient Bio Inc. (Seongnam-si, Republic of Korea). All the animal experimental procedures were reviewed and approved by the Animal Care Use Committee at Yonsei University (IACUC No. 2023-0226) and were performed according to the International Guide for the Care and Use of Laboratory Animals. The mice were housed in temperature- (20–24 °C) and humidity- (30–70%)controlled rooms under a 12 h light/dark cycle. The body weight and the changes in health condition were monitored weekly. The mice with a 20% peak weight loss or severe illness were euthanized. The nude mice were inoculated subcutaneously with 1 × 10^6^ of the U87MG and U87MG vIII 4.12 cells in the right shoulders under 2% isoflurane anesthesia.

### 2.11. Biodistribution

When the average U87MG and U87MG vIII 4.12 tumor size reached 300–400 mm^3^, the nude mice were injected with the ^68^Ga-NOTA-EGFRvIII aptamer (7.4–11.1 MBq, 500 pmol) via the tail vein under 2% isoflurane anesthesia and sacrificed 30 min and 60 min (n = 4 for each group) after injection. The animals were anesthetized with 5% isoflurane, and blood was harvested from the right ventricle of the heart, followed immediately by cervical dislocation. The major organs and tissues were collected and weighed. The radioactivity of each sample was measured using a 1470 automatic gamma counter (PerkinElmer–Wallac, Waltham, MA, USA). The radioactivity concentration is expressed as a percentage of the injected dose per gram of tissue (%ID/g). For each mouse, the radioactivity of the tissue samples was calibrated against a known aliquot of the injected activity.

### 2.12. PET Imaging

Small-animal PET images were obtained on an Inveon microPET scanner (Siemens, Knoxville, TN, USA). The tumor-bearing mice were injected with the ^68^Ga-NOTA-EGFRvIII aptamer (7.4–11.1 MBq, 500 pmol) via the tail vein under 2% isoflurane anesthesia. Static PET scans were performed 60 min after the injection with an acquisition time of 20 min. The images were reconstructed using a three-dimensional ordered subsets expectation maximization (3D-OSEM) algorithm. The region of interest was manually drawn over the tumors, and the ^68^Ga-NOTA-EGFRvIII aptamer uptake was quantified using the ASIPro software (version 6.2.5.0, Siemens).

### 2.13. Statistics

All the data are presented as the means ± standard deviation (SD). An unpaired Student’s *t*-test was performed using GraphPad Prism 5.0 (GraphPad Software, San Diego, CA, USA). Differences were considered significant at a *p*-value of less than 0.05.

## 3. Results

### 3.1. Expression of EGFRvIII in Human Glioma Cell Lines

**Figure 1:**
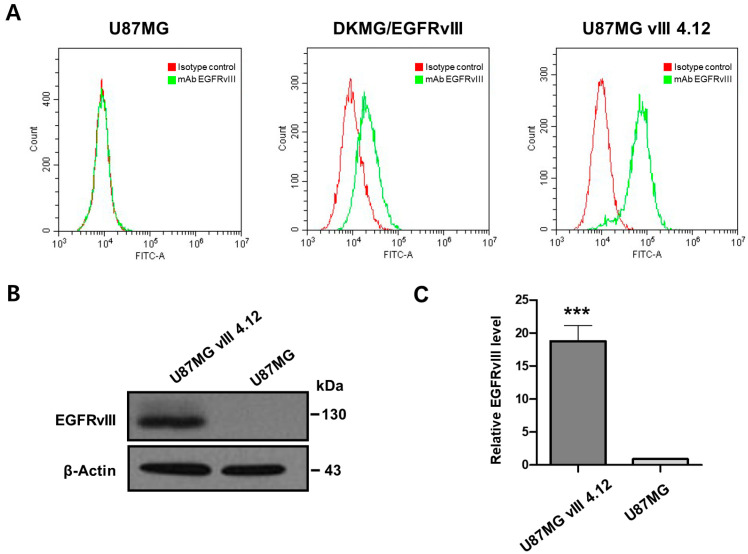
Evaluation of EGFRvIII expression in glioma cell lines. (**A**) EGFRvIII expression in the EGFRvIII-engineered U87MG cell lines (DKMG/EGFRvIII and U87MG vIII 4.12) and U87MG cells was analyzed by flow cytometry using a monoclonal antibody (mAb) against EGFRvIII. (**B**) The Western blot analysis of the EGFRvIII level in the U87MG vIII 4.12 and U87MG cells. β-Actin was used as an endogenous control. (**C**) The relative intensities of the EGFRvIII proteins were calculated by comparing them to the intensity of β-actin. The data are presented as the mean ± SD of three independent experiments. Statistical analysis was performed using a Student’s *t*-test. *** *p* < 0.001.

Human U87MG glioma cells have little to no EGFRvIII expression on their surface [21,22]. Thus, the EGFRvIII-engineered U87MG cell lines (DKMG/EGFRvIII and U87MG vIII 4.12) were utilized as the target cells for the EGFRvIII aptamer specificity. The expression of the EGFRvIII protein in human glioma cell lines was validated by flow cytometry using the anti-EGFRvIII antibody. Flow cytometry showed that the U87MG vIII 4.12 cells had the highest EGFRvIII expression on the cell surface, while no EGFRvIII expression was observed in the U87MG cells (Figure 1A).

The EGFRvIII protein expression was validated further by Western blot. Consistent with the flow cytometry results, the Western blot analysis showed that the band intensity of EGFRvIII expression in the U87MG vIII 4.12 cells was significantly higher than in the U87MG cells (*p* < 0.001) (Figure 1B).

### 3.2. EGFRvIII Aptamer Binds Specifically to EGFRvIII Expressing Cells

**Figure 2:**
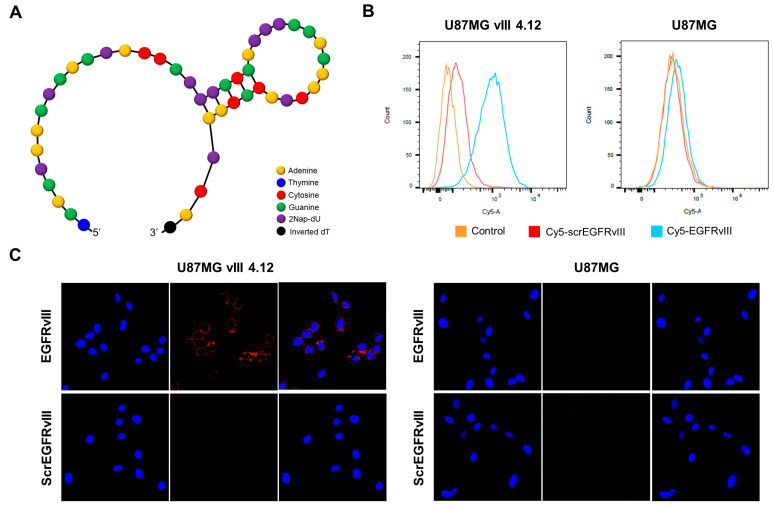
Characterization of the EGFRvIII aptamer in vitro. (**A**) The secondary structure of the EGFRvIII aptamer predicted by the RNAstructure web server according to the free energy minimization algorithm. (**B**) The flow cytometry analysis of the binding ability of the Cy5-labeled EGFRvIII aptamer (Cy5-EGFRvIII) against the EGFRvIII-positive U87MG vIII 4.12 cells and EGFRvIII-negative U87MG cells at 4 °C. The Cy5-labeled scrambled EGFRvIII (Cy5-ScrEGFRvIII) aptamer was used as the negative control. (**C**) The fluorescent microscopy of cell binding by the Cy5-EGFRvIII aptamer (red) to the U87MG vIII 4.12 cells and U87MG cells. The cells were stained with the Cy5-EGFRvIII aptamer at 4 °C, followed by washing and confocal microscopy imaging. The nuclei were stained with 4′,6-diamidino-2-phenylindole (DAPI, blue). Scale bar = 100 µm.

The EGFRvIII aptamer was selected using the Systematic Evolution of Ligands by EXponential enrichment (SELEX) technology; Figure 2A presents the secondary structure of the EGFRvIII aptamer. The binding affinity of the EGFRvIII aptamer for the EGFRvIII proteins was determined by measuring the K_d_. The K_d_ value of the EGFRvIII aptamer was 0.87 nM, showing the high affinity of the EGFRvIII aptamers for the EGFRvIII proteins. However, the EGFRvIII aptamer did not show binding ability to wild-type EGFR protein (Figure S1).

The specificity of the EGFRvIII aptamer to glioma cells was examined by flow cytometry using the 5′-end Cy5-labeled EGFRvIII aptamers. The Cy5-EGFRvIII aptamer was bound to U87MG vIII 4.12 at a higher extent than the Cy5-labeled scrambled EGFRvIII (ScrEGFRvIII) aptamer, whereas the Cy5-EGFRvIII aptamer showed no significant binding in the U87MG cells (Figure 2B). The specific binding of the EGFRvIII aptamer for each glioma cell was also correlated with anti-EGFRvIII antibody binding, indicating that the EGFRvIII aptamer can bind specifically to the EGFRvIII proteins expressed on the plasma membrane.

The binding of Cy5-EGFRvIII aptamer on the surface of the EGFRvIII-engineered U87MG cells was also examined using confocal microscopy. As shown in Figure 2C, the Cy5-EGFRvIII aptamer specifically colocalized at the membrane of U87MG vIII 4.12, whereas very little to no signal was observed on the U87MG cells. Cy5-ScrEGFRvIII did not bind to the U87MG vIII 4.12 and U87MG cells. Flow cytometry and confocal microscopy indicated that the EGFRvIII aptamer is highly specific for the EGFRvIII proteins.

### 3.3. Design and Validation of NOTA-EGFRvIII Aptamer

**Figure 3:**
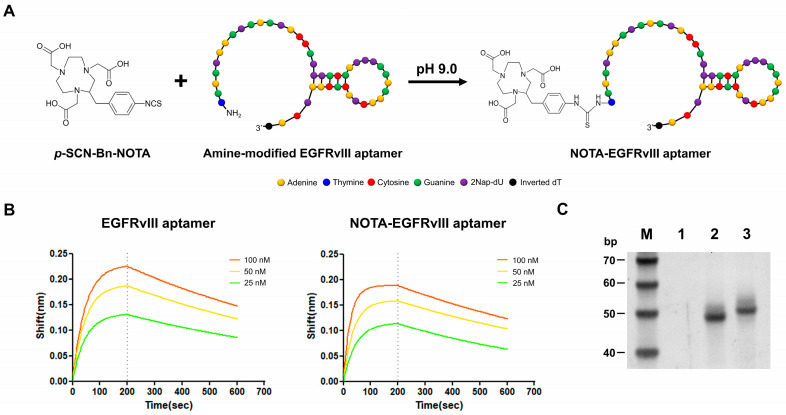
Characterization of the NOTA-EGFRvIII aptamer. (**A**) The schematic diagram of synthesis process for the preparation of the (1,4,7-triazanonane-1,4,7-triyl)triacetic acid (NOTA)-conjugated EGFRvIII (NOTA-EGFRvIII) aptamer. (**B**) The biolayer interferometry (BLI) data of the EGFRvIII aptamers and NOTA-EGFRvIII aptamers against various concentrations of EGFRvIII protein. (**C**) The evaluation of EGFRvIII aptamer conjugation with NOTA using urea/polyacrylamide gel electrophoresis. Lane M: 20/100 DNA ladder, Lane 1: empty vector, Lane 2: EGFRvIII aptamer, and Lane 3: NOTA-EGFRvIII aptamer.

The NOTA-conjugated EGFRvIII aptamer was prepared by conjugating an amine-functionalized EGFRvIII aptamer with *p*-SCN-Bn-NOTA using the protocol reported elsewhere (Figure 3A) [19]. The K_d_ value of the NOTA-EGFRvIII aptamer was 2.05 nM, indicating that NOTA conjugation did not significantly affect the binding affinity of the EGFRvIII aptamers (Figure 3B). The conjugation of the NOTA-EGFRvIII aptamer was confirmedwith urea-PAGE. The NOTA-EGFRVIII aptamers showed less electrophoretic mobility than the non-conjugated aptamers because of the increase in molecular weight (Figure 3C).

### 3.4. Radiosynthesis of ^68^Ga-NOTA-EGFRvIII Aptamer

**Figure 4:**
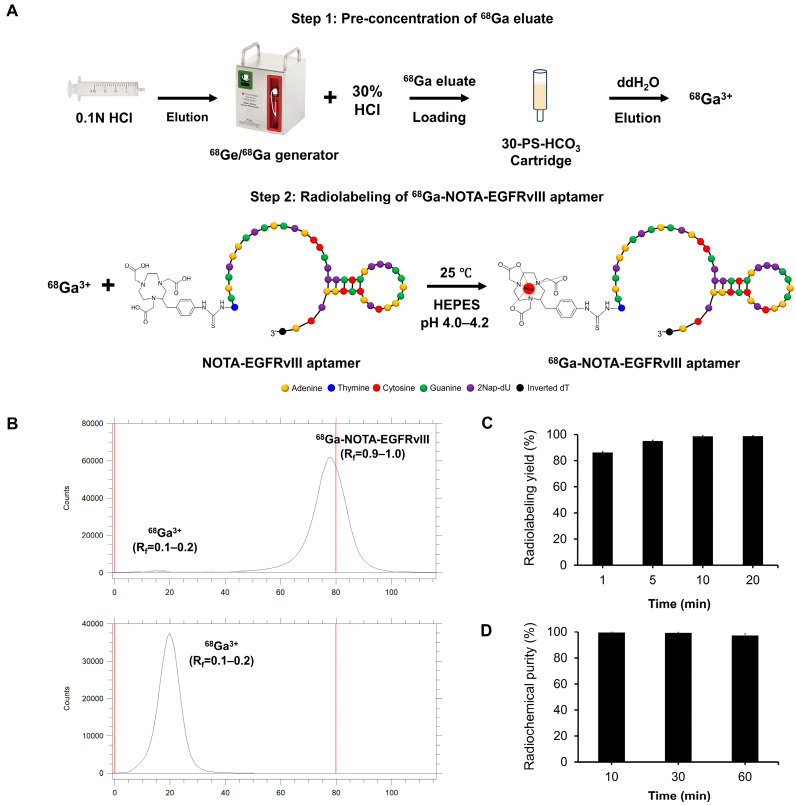
Radiolabeling of the NOTA-EGFRvIII aptamer with gallium-68. (**A**) The schematic diagram of the anionic purification of the ^68^Ga eluate and the synthesis of the ^68^Ga-NOTA-EGFRvIII aptamer. (**B**) The representative radio-TLC chromatograms of the ^68^Ga-NOTA-EGFRvIII (**upper** image) and free ^68^Ga^3+^ (**lower** image). R_f_: relative to the front. (**C**) The influence of the reaction time on the ^68^Ga-radiolabeling of the NOTA-EGFRvIII aptamer. The radiolabeling was analyzed by radio-TLC. (**D**) The in vitro stability of the ^68^Ga-NOTA-EGFRvIII aptamer was investigated in PBS buffers at 37 °C. The radiochemical purity of the ^68^Ga-NOTA-EGFRvIII aptamer was monitored by radio-TLC for up to 60 min. The data represent the mean ± SD from three independent experiments.

The ^68^Ga-labeled NOTA-EGFRvIII (^68^Ga-NOTA-EGFRvIII) aptamer was prepared using a preconcentration-based labeling method on an anion-exchange cartridge Chromafix 30-PS-HCO_3_ (Figure 4A) [20]. The 30-PS-HCO_3_ cartridge could trap 95.4 ± 1.5% of the activity from the ^68^Ge/^68^Ga-generator eluate. In addition, the recovery yield of ^68^Ga^3+^ from the 30-PS-HCO_3_ cartridge was 78 ± 4% (n = 10) with 0.3 mL of ddH_2_O.

The NOTA-EGFRvIII aptamer was buffered with 0.25 M sodium acetate to a pH of 4.0–4.2 and labeled with preconcentrated ^68^Ga^3+^ at room temperature. The effect of reaction time on radiochemical yield was monitored by radio-TLC (Figure 4B). The radiolabeling yield for the ^68^Ga-NOTA-EGFRvIII aptamer at 1, 5, 10, and 20 min time intervals was determined to be 86.1 ± 0.8%, 95.0 ± 0.8%, 98.5 ± 0.6%, and 98.7 ± 0.5%, respectively (Figure 4C). Extending the incubation time to 20 min did not improve the labeling efficiency, and the optimal reaction time was 10 min. ^68^Ga^3+^ showed no binding to the unconjugated EGFRvIII aptamer at pH 4.0 (Figure S2). Under optimized conditions, the radiolabeling of the ^68^Ga-NOTA-EGFRvIII aptamer was performed with a high radiochemical yield and purities > 98% (n = 10) and a molar activity of 18.5 ± 3.7 MBq/nmol at the end of synthesis (EOS). The ^68^Ga-NOTA-EGFRvIII aptamer was used for in vivo applications without further purification owing to the high radiochemical purity. The ^68^Ga-NOTA-EGFRvIII aptamer showed excellent stability in PBS up to 60 min at 37 °C (>98%) (Figure 4D).

### 3.5. Ex Vivo Biodistribution of ^68^Ga-NOTA-EGFRvIII Aptamer

**Figure 5:**
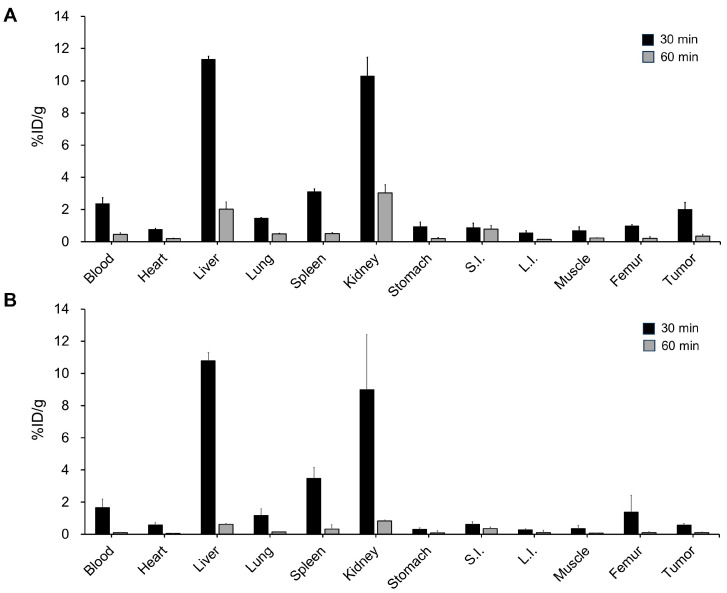
Ex vivo biodistribution of the ^68^Ga-NOTA-EGFRvIII aptamer in the mice bearing subcutaneous U87MG vIII 4.12 tumors. The uptake levels of the blood, heart, liver, lung, spleen, stomach, muscle, small intestine (S.I.), large intestine (L.I.), bone, and tumor at 30 min and 60 min after the tail vein injection of the ^68^Ga-NOTA-EGFRvIII aptamer in the mice bearing subcutaneous (**A**) EGFRvIII-positive U87MG vIII 4.12 tumors or (**B**) EGFRvIII-negative U87MG tumors. The whole biodistribution profiles are shown in the Supplementary Material. The data are expressed as a percentage of injected dose per gram tissue (%ID/g ± SD, n = 4 at each time point).

Biodistribution studies were performed in the U87MG vIII 4.12 tumor-bearing mice to evaluate the pharmacokinetic properties of ^68^Ga-NOTA-EGFRvIII aptamer in vivo. The blood levels of the ^68^Ga-NOTA-EGFRvIII aptamer decreased significantly between 30 min and 60 min post-injection from 2.37 ± 0.37 %ID/g to 0.45 ± 0.11 %ID/g (Figure 5A). The kidney showed the highest initial accumulation of the ^68^Ga-NOTA-EGFRvIII aptamer with an uptake of 10.30 ± 1.16 %ID/g at 30 min post-injection. The accumulation decreased to 3.03 ± 0.51 %ID/g at 60 min. The biodistribution studies also showed significantly increased radioactivity in the liver (11.34 ± 0.18 %ID/g at 30 min and 2.03 ± 0.45 %ID/g at 60 min), suggesting that the ^68^Ga-NOTA-EGFRvIII aptamer is also excreted via the hepatobiliary pathways. The tumor uptake of the ^68^Ga-NOTA-EGFRvIII aptamer in the U87MG vIII 4.12 tumor-bearing mice was 0.35 ± 0.11 %ID/g at 60 min post-injection, which was significantly higher than in the U87MG tumor-bearing mice (0.11 ± 0.03 %ID/g, *p* < 0.05) (Figure 5B). The muscle, heart, lung, spleen, and bone uptake were significantly lower than that of the tumor at all time points.

### 3.6. In Vivo PET Imaging of ^68^Ga-NOTA-EGFRvIII Aptamer

**Figure 6:**
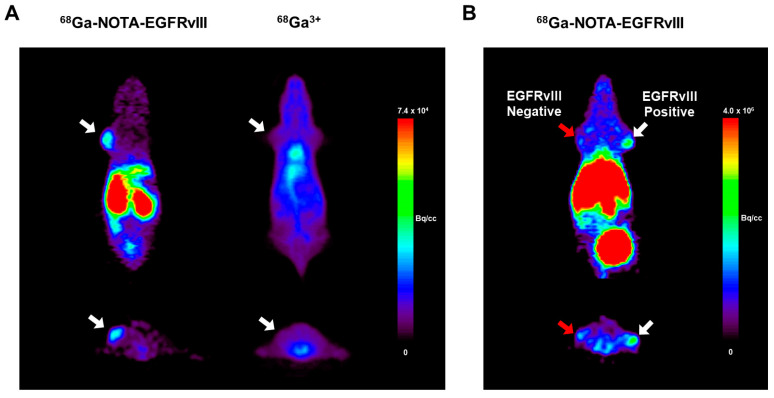
MicroPET imaging of the ^68^Ga-NOTA-EGFRvIII aptamer in the mice bearing subcutaneous U87MG vIII 4.12 tumors. (**A**) Representative coronal and transverse microPET images at 60 min after a tail vein injection of the ^68^Ga-NOTA-EGFRvIII aptamer (**left**) or ^68^Ga^3+^ (**right**). The white arrows indicate the location of the tumors. (**B**) The representative whole-body microPET images of the ^68^Ga-NOTA-EGFRvIII aptamer in the mice bearing the EGFRvIII-positive U87MG vIII 4.12 (**right**) and EGFRvIII negative U87MG tumors (**left**). The white arrow indicated the location of the U87MG vIII 4.12 tumors, and the red arrow indicated the region of the U87MG tumors.

The whole-body microPET imaging of the ^68^Ga-NOTA-EGFRvIII aptamer was carried out in the Balb/c nude mice bearing U87MG vIII 4.12 tumors. The U87MG vIII 4.12 tumor was clearly visible at 60 min post-injection in the U87MG vIII 4.12 tumor-bearing mice. However, there was no uptake of ^68^Ga^3+^ at the tumor site. In accordance with the ex vivo biodistribution data, the PET image of the ^68^Ga-NOTA-EGFRvIII aptamer showed high uptake in the kidneys (Figure 6A).

The in vivo target ability of the ^68^Ga-NOTA-EGFRvIII aptamer was evaluated by establishing EGFRvIII positive (U87MG vIII 4.12) and EGFRvIII negative (U87MG) xenografts in the opposite upper shoulder of the athymic nude mice. The MicroPET imaging showed that the uptake of the ^68^Ga-NOTA-EGFRvIII aptamer was significantly high in the EGFRvIII positive tumors compared to that of the EGFRvIII negative tumors at 60 min post-injection, which was consistent with the biodistribution profiles (Figure 6B). No specific uptake of the ^68^Ga-NOTA-EGFRvIII aptamer was observed in the brain (Figure S3).

## 4. Discussion

EGFRvIII is the most common point mutation in the extracellular domain of EGFR [23]. EGFRvIII is a promising target in cancer detection because of its strong expression in cancer cells but not in normal cells [24,25]. Despite this, few studies have reported the in vivo PET imaging of EGFRvIII using aptamer. This study tested the feasibility of a radioisotope-labeled EGFRvIII aptamer as an imaging agent for EGFRvIII-expressing tumors. The bifunctional chelator NOTA was conjugated to the EGFRvIII aptamer and labeled with ^68^Ga to visualize and evaluate the EGFRvIII expression in mouse tumor models. The ex vivo biodistribution studies revealed the highly selective targeting of the ^68^Ga-NOTA-EGFRvIII aptamer in the EGFRvIII positive U87MG vIII 4.12 tumor-bearing mice. Moreover, the in vivo PET images showed that the tumor uptake of the ^68^Ga-NOTA-EGFRvIII aptamer was significantly higher in the U87MG vIII 4.12 tumors than in the EGFRvIII negative U87MG tumors.

Oligonucleotides, including aptamers, antisense oligonucleotides, and small interfering RNA, have been developed and investigated widely as molecular imaging probes to monitor various cellular processes and therapeutic efficacy or to assess the target specificity [26]. Nuclear imaging is an attractive and powerful molecular imaging tool owing to its high sensitivity, accurate quantification, limitless penetration depth, and good spatial resolution [27,28]. Various radioisotopes, including fluorine-18, technetium-99m, copper-64, iodine-125, indium-111, and gallium-68 can be labeled with oligonucleotides for nuclear imaging [29]. Among these, ^68^Ga (T_1/2_ = 68 min) is the most promising positron-emitting radioisotope readily obtained using a ^68^Ge/^68^Ga generator system. The radiolabeling of biomolecules with ^68^Ga requires a bifunctional chelator, including 1,4,7,10-tetraazacyclododecane-1,4,7,10-tetreaacetic acid (DOTA), Tris(hydroxypyridinone) (THP), triazacyclononane-phosphinate (TRAP), and NOTA [30]. Schlesinger et al. conjugated DOTA to L-RNA oligonucleotide via the N-hydroxysuccinimide coupling reaction and radiolabeled it with gallium-68 and yttrium-86 [31]. Gijs et al. optimized the conjugation method of NOTA to DNA oligonucleotide through a thiol/maleimide reaction for ^68^Ga radiolabeling [19]. NOTA is a commonly used macrocyclic bifunctional chelator for radiobioconjugates because of its high thermodynamic stability and labeling efficiency at room temperature [32,33]. In the present study, NOTA was selected as the chelator for the nuclear imaging of an aptamer because the ^68^Ga-radiolabeling of NOTA is faster and more efficient under milder conditions at room temperature than DOTA.

EGFRvIII is overexpressed in approximately 28–30% of glioblastomas, suggesting that EGFRvIII is a potential target for cancer diagnosis and therapy [3]. In this study, SELEX was conducted to select the DNA aptamers against the EGFRvIII proteins. The EGFRvIII aptamer can bind specifically to the EGFRvIII-expressing U87MG vIII 4.12 cells and be internalized into the glioma cells. The co-localization of the EGFRvIII aptamer was also confirmed with the late endosomal and lysosomal marker, suggesting that the EGFRvIII aptamer was internalized into the glioma cells via the endocytic pathway. After confirming the cell binding specificity and internalization of aptamer, macrocyclic bifunctional chelator NOTA was conjugated to the EGFRvIII aptamer for nuclear imaging. The NOTA-EGFRvIII aptamer was prepared through the amine/isothiocyanate conjugation reaction between the *p*-SCN-Bn-NOTA and amine-modified aptamer. A BLI assay confirmed that the NOTA-EGFRvIII aptamer displayed a comparable K_d_ value to the EGFRvIII aptamer.

The preconcentration and purification of the ^68^Ga eluate using a disposable cartridge is a widely used method for ^68^Ga-radiolabeling to reduce the volume and HCl concentration of the ^68^Ga eluate and to maximize the radiolabeling yields [34,35]. In the present study, the ^68^Ga eluate was preconcentrated using an anion-exchange cartridge to increase the labeling efficiency of the NOTA-EGFRvIII aptamer. The radiolabeling condition was optimized and showed that the high radiochemical yield of the ^68^Ga-NOTA-EGFRvIII aptamer was achieved within 10 min at room temperature and pH 4.

Based on the ex vivo biodistribution studies, the ^68^Ga-NOTA-EGFRvIII aptamer displayed rapid excretion mainly via the renal system, probably due to the small molecular weight and hydrophilicity of aptamers [36,37,38]. The liver and small intestine also showed relatively high accumulation at 60 min post-injection compared to the other organs, suggesting that the ^68^Ga-NOTA-EGFRvIII aptamer is mainly excreted through the kidney at the early time point and then excreted via the hepatobiliary pathways. The ex vivo biodistribution of the U87MG vIII 4.12 tumor-bearing mice displayed a significantly higher uptake of the ^68^Ga-NOTA-EGFRvIII aptamer in the EGFRvIII positive U87MG vIII 4.12 tumors than in the EGFRvIII negative U87MG tumors.

PET also showed that the ^68^Ga-NOTA-EGFRvIII aptamer was excreted through the renal and hepatobiliary routes. The in vivo tumor-targeting specificity was evaluated in the athymic nude mice bearing paired U87MG vIII 4.12 and U87MG xenografts in the opposite upper shoulder. The microPET image revealed the high retention rate of the ^68^Ga-NOTA-EGFRvIII aptamer in the EGFRvIII positive tumors, but only low levels of uptake in the EGFRvIII negative tumors at 60 min post-injection, indicating that the ^68^Ga-NOTA-EGFRvIII aptamer selectively targets EGFRvIII. These results demonstrate that the ^68^Ga-NOTA-EGFRvIII aptamer may be used as an in vivo PET imaging probe for EGFRvIII-expressing glioblastomas.

There were some limitations in our study. We just evaluated the targeting ability of the ^68^Ga-NOTA-EGFRvIII aptamer in the subcutaneous U87MG vIII 4.12 glioblastoma xenografted mice model, not the orthotopic brain tumor model. In the subcutaneous U87MG vIII 4.12 glioblastoma xenografted model, the ^68^Ga-NOTA-EGFRvIII aptamer was not able to cross the blood/brain barrier (BBB) in the microPET image (Figure S3). However, it is known that BBB disruption could occur in a variety of pathological conditions, including brain tumors. Therefore, we will have to investigate whether the ^68^Ga-NOTA-EGFRvIII aptamer could penetrate through the BBB in an orthotopic glioblastoma mice model in further studies.

## Data Availability

All the data are contained within the article.

## Supplementary Materials

The following supporting information can be downloaded at: https://www.mdpi.com/article/10.3390/pharmaceutics16060814/s1, Figure S1: Biolayer interferometry data of the EGFRvIII aptamers against various concentrations of wild-type EGFR protein.; Figure S2: Representative radio-TLC chromatograms of the unconjugated EGFRvIII aptamer reacted with ^68^Ga^3+^ (upper image) and free ^68^Ga^3+^ (lower image). R_f_: relative to the front.; Figure S3. Representative whole-body microPET images of ^68^Ga-NOTA-EGFRvIII aptamer in mice bearing U87MG vIII 4.12 and U87MG tumors. The white arrow indicated the location of the brain.
