# Supplementary material for: In Vivo Evaluation of 68Ga-Labeled NOTA-EGFRvIII Aptamer in EGFRvIII-Positive Glioblastoma Xenografted Model
Source: Pharmaceutics. 2024 Jun 16;16(6):814. doi: 10.3390/pharmaceutics16060814 (PMC11207964; doi:10.3390/pharmaceutics16060814)
Supplement: Supplementary file 1 [file pharmaceutics-16-00814-s001.zip › pharmaceutics-3018222-supplementary.pdf]

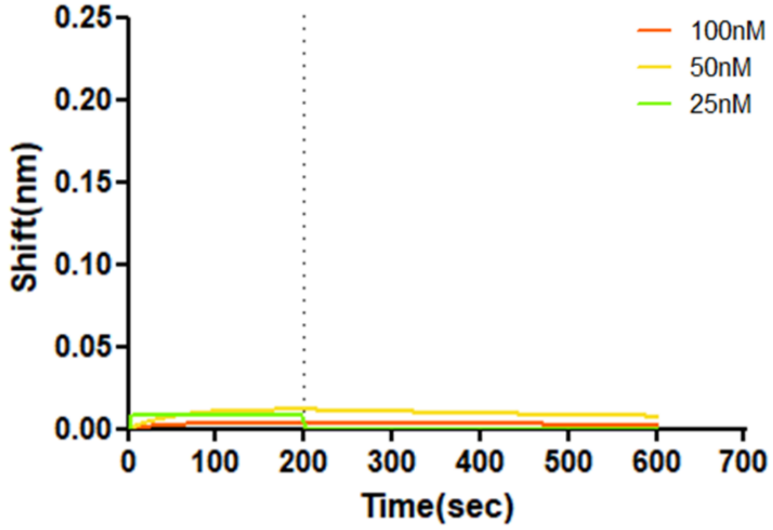

Figure S1. Biolayer interferometry data of the EGFRvIII aptamers against various concentrations of wild-type EGFR protein.

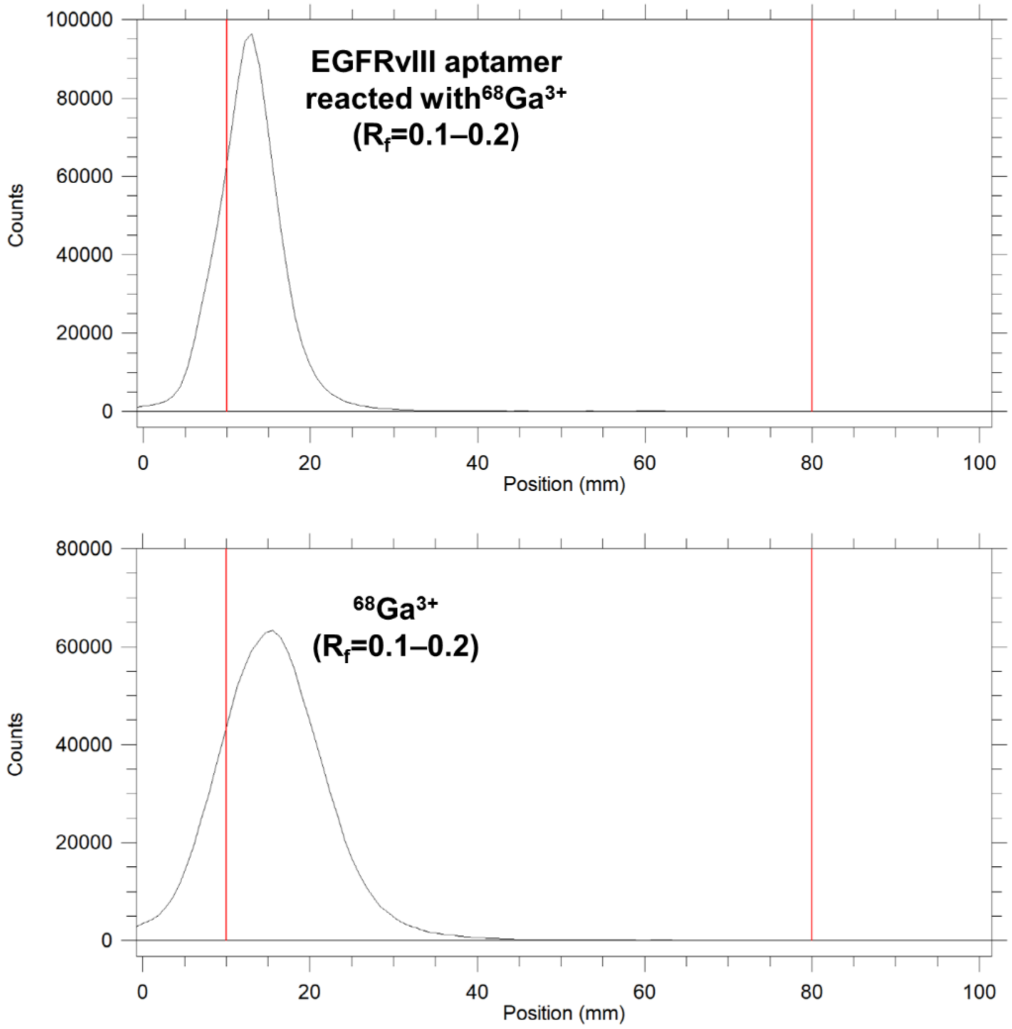

Figure S2. Representative radio-TLC chromatograms of the unconjugated EGFRvIII aptamer reacted with $^{68}Ga^{3+}$ (upper image) and free $^{68}Ga^{3+}$ (lower image). R_f: relative to the front.

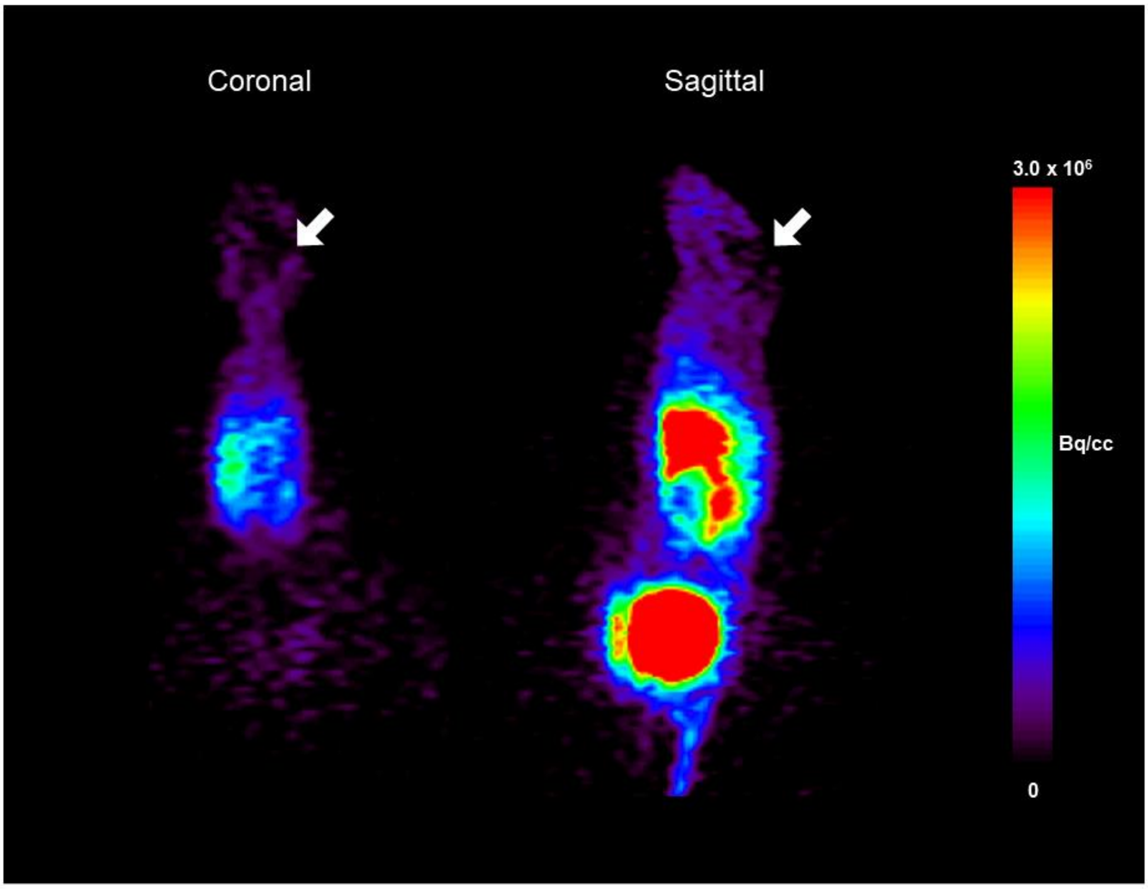

Figure S3. Representative whole-body microPET images of ^{68}Ga-NOTA-EGFRvIII aptamer in mice bearing U87MG vIII 4.12 and U87MG tumors. The white arrow indicated the location of the brain.
